# Prosthetic valve endocarditis caused by *Propionibacterium* species: a national registry-based study of 51 Swedish cases

**DOI:** 10.1007/s10096-017-3172-8

**Published:** 2018-01-29

**Authors:** Fredrik Lindell, Bo Söderquist, Kristina Sundman, Lars Olaison, Jan Källman

**Affiliations:** 1 Section of Infectious Diseases, Department of Medical Sciences, Uppsala University, Uppsala, Sweden; 2 Department of Infectious Diseases, Uppsala University Hospital, 751 85 Uppsala, Sweden; 3 School of Medical Sciences, Faculty of Medicine and Health, Örebro University, Örebro, Sweden; 4 Department of Laboratory Medicine, Clinical Microbiology, Örebro University Hospital, 701 85 Örebro, Sweden; 5 Department of Infectious Diseases, Institute of Biomedicine, University of Gothenburg, Gothenburg, Sweden; 6 Swedish Registry of Infective Endocarditis, Swedish Society of Infectious Diseases, Gothenburg, Sweden; 7 Department of Infectious Diseases, Sahlgrenska University Hospital, 416 85 Göteborg, Sweden; 8 Department of Infectious Diseases, Faculty of Medicine and Health, Örebro University, Örebro, Sweden; 9 Department of Infectious Diseases, Örebro University Hospital, 701 85 Örebro, Sweden

## Abstract

*Propionibacterium* spp. are a rare cause of infective endocarditis (IE). The diagnosis is difficult because the bacteria are slow-growing and growth in blood cultures is often misinterpreted as contamination from the skin flora. The aim of this study was to describe all cases of *Propionibacterium* spp. endocarditis in the Swedish national registry of IE. The registry was searched for all cases of IE from 1995 to 2016 caused by *Propionibacterium* spp. Data concerning clinical characteristics, treatment, and outcome were registered. A total of 51 episodes of definitive prosthetic valve endocarditis (PVE) caused by *Propionibacterium* spp. were identified, comprising 8% of cases of PVE during the study period. Almost all cases (*n* = 50) were male. The median time from surgery to diagnosis of IE was 3 years. Most patients were treated mainly with beta-lactams, partly in combination with aminoglycosides. Benzyl-penicillin was the most frequently used beta-lactam. A total of 32 patients (63%) underwent surgery. Overall, 47 patients (92.1%) were cured, 3 (5.9%) suffered relapse, and 1 (2.0%) died during treatment. IE caused by *Propionibacterium* spp. almost exclusively affects men with a prosthetic valve and findings of *Propionibacterium* spp. in blood cultures in such patients favors suspicion of a possible diagnosis of IE. In patients with prosthetic valves, prolonged incubation of blood cultures up to 14 days is recommended. The prognosis was favorable, although a majority of patients required cardiac surgery during treatment. Benzyl-penicillin should be the first-line antibiotic treatment option for IE caused by *Propionibacterium* spp.

## Introduction

Infective endocarditis (IE) is a serious condition, requiring long-term antibiotic treatment and, occasionally, surgical intervention. In Sweden, the mortality of IE following treatment is among the lowest in the world, being approximately 10% [1]. Prosthetic valve endocarditis (PVE) is often more complicated, regarding both diagnosis and treatment; a European study and a global study reported in-hospital mortality rates of 15% and 25%, respectively [2, 3]. PVE can be divided into early and late onset, with early onset presenting during the first 12 months after surgery and late onset more than 12 months after surgery [4].

*Propionibacterium acnes* is commonly associated with acne vulgaris, but is also known to sometimes cause serious infections, often when implanted material such as prosthetic joints and neurosurgical shunts are present [5, 6]. *Propionibacterium acnes* has been shown to produce biofilm, and the capability for biofilm production seems to be characteristic of invasive isolates [7]. *Propionibacterium acnes* is a rare cause of IE, comprising approximately 0.3% of all IE cases [8]. The diagnosis of these infections may be difficult, since the bacteria are slow-growing and findings in blood cultures are often interpreted as contamination from the skin flora [5, 9].

Previous studies of IE caused by *P. acnes* are scarce, but show that IE caused by *P. acnes* is a disease predominantly affecting middle-aged men, with presence of prosthetic valves or pacemakers as obvious risk factors [8–10]. The reported mortality in IE due to *P. acnes* endocarditis is 13–24% [8]. There are currently no guidelines available for the treatment of IE caused by *P. acnes* or other *Propionibacterium* spp.

The aim of this nationwide study was to describe the clinical characteristics, management, and outcome of IE caused by *Propionibacterium* spp. in Sweden.

## Method

In 1995, the Swedish Society for Infectious Diseases introduced a Swedish national registry of IE (the SRIE). All 30 departments of infectious diseases (ID) in Sweden have participated in the registry since its inception. These ID departments have regional responsibility for the care of patients with severe infections, and patients requiring acute surgery for IE are, in most cases, treated in ID departments during the pre- and/or postoperative period.

One aim of the registry was to create a consistent diagnostic and therapeutic approach to patients with fever without definite non-cardiac origin. All cases are reported on a standardized questionnaire at the time of discharge and a second questionnaire after follow-up (mean: 3 months after treatment). Data regarding gender, age, comorbidity, risk factors, heart failure, affected valve, presence of prosthetic valve, type of prosthetic valve, time since surgery, and presence of other implantable cardiac devices such as a pacemaker or intracardiac defibrillator (ICD) are collected. Clinical characteristics at presentation such as fever, new murmur, and vascular phenomena are recorded. The origin of the etiologic agent is verified using, for example, blood cultures, cultures from valves during surgery, and polymerase chain reaction (PCR) from tissue samples of valves. Information about findings on echocardiography, delay from onset of symptoms to start of treatment, antibiotic treatment, need for surgery, and treatment outcome is also included.

During the 22-year study period (1995–2016), 7734 adult episodes of IE were registered, corresponding to an estimated incidence of 4.0 cases/100,000 inhabitants/year. The SRIE is estimated to cover approximately 75% of all hospital-treated episodes in Sweden with a diagnosis of IE [11]. During 1995–2007, cases were reported on paper questionnaires, and in 2008, an internet-based reporting system was instituted. Data regarding diabetes, year of previous valve surgery, and type of valve prosthesis were not available for the first time period.

In the present study, we used the SRIE to identify cases of IE with *Propionibacterium* spp.

### Data availability

The datasets analyzed during the current study are not publicly available due to individual privacy but are available from the corresponding author on reasonable request.

## Results

**Chart 1:**
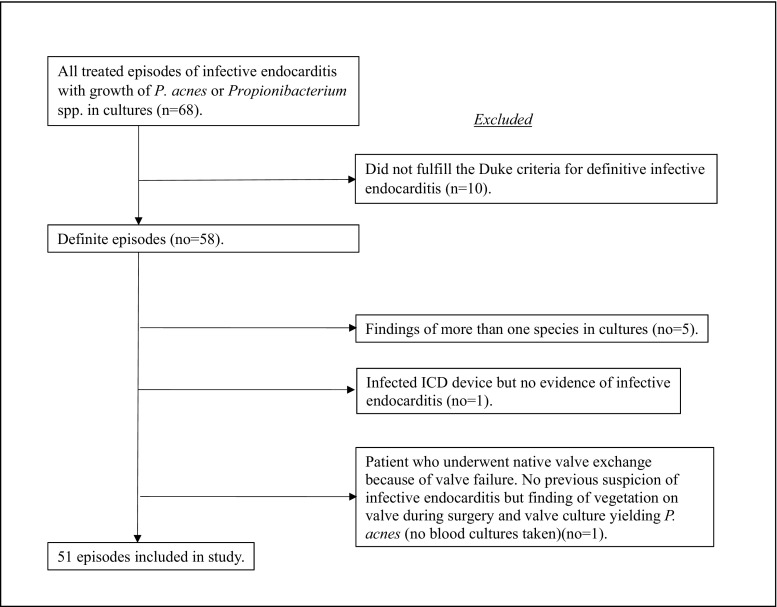
Reasons for exclusion from the study

The SRIE included 5909 definite episodes of IE according to the modified Duke criteria [12] from 1995 to 2016; 2953 episodes in 1995–2007 and 2956 episodes in 2008–2016. *Propionibacterium acnes* or *Propionibacterium* spp. were identified in 58 of the definite episodes; five of these cases had cultures with growth of two bacterial species [*P. acnes* + *Staphylococcus aureus*, *P. acnes* + coagulase-negative staphylococci (*n* = 3) or *P. acnes* + *Streptococcus mitis*] and were excluded. Two additional cases were also excluded: one patient with an infected ICD and no proof of infection on the valves and one patient who underwent valve surgery on a native valve because of valve failure and was found to have a vegetation on the valve with growth of *P. acnes* in valve culture; however, no blood cultures were taken (Chart 1). Another ten cases, reported as possible endocarditis, were not included in the study. All remaining cases were regarded as prosthetic valve infections.

During the period 2008–2016, with complete data regarding prosthesis implantations, 379 patients in the registry had a PVE. The 32 cases of *Propionibacterium* spp. endocarditis represented 8% of the total cases of PVE during this period.

A majority of cases were male (50/51; 98%). The only female patient had *P. avidum* in 10/10 blood cultures and a prosthetic aortic valve.

All patients had cultures with growth of *P. acnes* or *Propionibacterium* spp. either from blood cultures, from extracted valves, or from both. Microbiological diagnostics were performed at each individual County Department of Clinical Microbiology. The *Propionibacterium* spp. isolates were characterized according to routine laboratory procedures, such as indole and catalase tests and microscopic examination. Species determination was also performed by matrix-assisted laser desorption/ionization time-of-flight mass spectrometry (MALDI-TOF MS) after it was introduced. Data concerning antimicrobial susceptibility were not included in the registry.

The median time from last valve surgery to infection was 3 years (range: 0–22) in the later time period; data were not available in the older material.

The median patient delay (time from onset of symptoms to first contact with a physician) was 12 days (range: 0–139), and the median doctor delay (time from first physician contact to start of empiric or specific endocarditis treatment) was 2 days (range: 0–93).

In the majority of the episodes (42/51, 82%), a transesophageal echocardiography was performed, while seven patients underwent transthoracic echocardiography only. Two patients who did not undergo echocardiography were both operated with a prosthetic valve exchange due to valve dysfunction. During surgery, vegetation(s) on the valve were found, with growth of *P. acnes* in cultures of the valve.

Positive blood cultures were found in 44/51 episodes (86%). Six patients displayed positive cultures for *Propionibacterium* spp. on the extracted valve despite negative blood cultures, and ten patients were positive in both blood and valve cultures. PCR on the extracted valve material was positive for *P. acnes* in four cases, all of which were positive in blood cultures, but two had negative valve cultures. Identification to the species level showed *P. acnes* in 38 cases, *P. avidum* in one, and *Propionibacterium* spp. in 12.

The patient characteristics and clinical details are summarized in Table 1.

**Table 1:** The characteristics of 51 patients with definite infective prosthetic valve endocarditis (PVE) caused by *Propionibacterium* spp. in the Swedish national registry (1995–2016)

	Total (*n* = 51)		1995–2007 (*n* = 19)	2008–2016 (*n* = 32)	*p*-Value^b^	Surgically treated (*n* = 32)		Medically treated (*n* = 19)		*p*-Value^c^
	No.	%				No.	%	No.	%	
Sex										
Female	1	2.0	0	1		1	3.1	0	0.0	
Male	50	98.0	19	31		31	96.9	19	100	
Age, years, median (range)	64 (26–88)		58 (37–85)	70 (26–88)		63 (34–77)		74 (26–88)		
Predisposing factors										
Congenital heart failure			No data	1		No data		No data		
Rheumatic heart disease			No data	1		No data		No data		
History of endocarditis	10	19.6	7	3	0.027	4	12.5	6	31.6	0.146
Prosthetic valve	51	100	19	32	1	32	100	19	100	1
Mechanical valve prosthesis			No data	12		8	25.0	No data		
Biological valve prosthesis			No data	19		11	34.4	No data		
Type of prosthesis not registered	20	39.2	19	1		13	40.6	7	36.8	1
PM	6	11.8	1	5	0.392	4	12.5	2	10.5	1
ICD	2	3.9	0	2	0.523	2	6.3	0	0.0	0.523
No foreign material (prosthesis/PM/ICD)	0	0.0	0	0		0	0.0	0	0.0	
Years since valve surgery, median (range)			No data	3 (0–22)		No data				
Clinical characteristics										
Fever	38	74.5	17	21	0.096	22	68.8	16	84.2	0.323
Vascular phenomena	12	23.5	5	7	0.743	4	12.5	8	42.1	0.037
New heart murmur	5	9.8	1	4	0.639	3	9.4	2	10.5	1
Echocardiography findings										
Vegetation	33	64.7	9	24	0.070	18	56.3	15	78.9	0.135
Myocardial abscess	13	25.5	4	9	0.743	11	34.4	2	10.5	0.096
Valvular leakage	20	39.2	9	11	0.389	17	53.1	3	15.8	0.016
Prosthetic valve dehiscence	10	19.6	6	4	0.146	10	31.3	0	0.0	0.008
Other finding compatible with endocarditis	7	13.7	1	6	0.236	5	15.6	2	10.5	0.670
Valve involved										
Aortic valve^a^	47	92.2	18	29	1	30	93.8	17	89.5	0.623
Mitral valve^a^	4	7.8	1	3	1	2	6.3	2	10.5	0.623
Microbiology										
Positive blood cultures	44	86.3	15	29	0.402	25	78.1	19	100	0.037
Positive valve cultures	16	50.0				16	50.0	Not applicable		
Positive valve PCR	4	12.5				4	12.5	Not applicable		

^a^Aortic, mitral, tricuspid, and pulmonic valves (one patient), aortic and mitral valves (one patient)

^b^*p*-Values using two-tailed Fisher’s exact test comparing patients in the period 1995–2007 with those in the period 2008–2016

^c^*p*-Values using two-tailed Fisher’s exact test comparing patients who underwent surgery with patients receiving antibiotic treatment only

### Antibiotic treatment

All patients in the study received antibiotic treatment. Treatment regimens were determined for 48 episodes, 44 of which were declared cured (Table 2). The median duration of treatment was 42 days (range: 18–117). A majority were treated with beta-lactam antibiotics (*n* = 43), often initially in combination with aminoglycosides (*n* = 37). The median duration of treatment with beta-lactams was 38 days (range: 9–71), with the addition of aminoglycosides for a median of 14 days. Twenty-six patients received treatment with vancomycin part of the time in combination with an aminoglycoside. The most frequently used beta-lactams were benzyl-penicillin (*n* = 40), followed by cefuroxime, ceftriaxone, cefotaxime, and carbapenems. Part of the time, seven patients were treated with rifampicin and nine with clindamycin. Data concerning the dosage of antibiotics were not included in the registry.

**Table 2:** Antibiotic treatment, type, and duration in 44 cured patients with prosthetic valve endocarditis (PVE) caused by *Propionibacterium* spp.^a^

Type of antibiotic	Total (*n* = 44)		Surgically treated (*n* = 28)		Medically treated (*n* = 16)	
	Treatment duration, days, median (range)	Patients, no.	Treatment duration, days, median (range)	Patients, no.	Treatment duration, days, median (range)	Patients, no.
Total antibiotic treatment	42 (18–117)	44	41 (18–80)	28	42 (28–117)	16
Beta-lactam	38 (9–71)	43	33 (9–71)	27	41 (19–47)	16
- PcG	33 (16–61)	40	33 (16–61)	26	38 (24–43)	14
- Cefuroxime/ceftriaxone/cefotaxime	7 (2–40)	14	8 (2–40)	9	13 (2–29)	5
Vancomycin	9 (2–40)	26	9 (2–37)	17	10 (5–42)	9
Aminoglycoside	14 (2–41)	37	10 (2–41)	24	14 (2–39)	13
Clindamycin	15 (3–80)	9	14 (3–42)	7	48 (15–80)	2
Rifampicin	19 (7–43)	7	10 (7–43)	3	24 (11–32)	4

^a^Excluded are one patient who died after 18 days of treatment, one patient with relapse 12 months post-treatment, and two episodes for one patient with two relapses 10 and 15 months post-treatment. Three patients had no data available

### Surgical treatment

Surgery during index hospitalization was performed in 32 episodes (63%). The median time to surgery was 5 days after commencing antibiotic treatment (range: 0–43). A comparison of the time periods 1995–2007 and 2008–2016 showed that surgery was performed sooner (median 3 vs. 16 days) and in older patients (median 64 vs. 58 years) during the second period.

Valve exchange was performed in 29 cases, valvular plastics in two, and in one case, the type of surgery was not registered.

### Outcome

Cure was achieved in 47/51 cases (92.2%). One patient (2.0%) died during treatment; a 75-year-old man with an infected aortic homograft who underwent surgical treatment on day 16 and succumbed 3 days later due to sepsis and heart decompensation (Table 3).

**Table 3:** Complications and outcome in 51 episodes of prosthetic valve endocarditis (PVE) caused by *Propionibacterium* spp.

Complication	Total (*n* = 51)		Surgically treated (*n* = 32)		Medically treated (*n* = 19)		*p*-Value^a^
	No.	%	No.	%	No.	%	
Cardiac failure	13	25.4	11	34.4	2	10.5	0.096
Cerebral involvement	9	17.6	4	12.5	5	26.3	0.266
Systemic embolism	6	11.8	4	12.5	2	10.5	1
Paravalvular abscess	13	25.4	11	34.4	2	10.5	0.096
In-hospital mortality	1	2.0	1	3.1	0	0	1
Relapse	3^b^	5.9	0	0	3^b^	15.8	0.046

^a^*p*-Value using two-tailed Fisher’s exact test comparing outcome in episodes treated surgically vs. antibiotic treatment only

^b^Three relapse episodes in total, with one patient suffering two relapses

Of the 19 episodes treated conservatively with antibiotics, 16 patients (84.2%) were declared cured and three relapse episodes (in two patients) were diagnosed (5.9%). A 76-year old man who had received a biological aortic valve prosthesis 3 years previously was first treated conservatively with 6 weeks of benzyl-penicillin in combination with aminoglycoside for 3 weeks. Twelve months later, a relapse was diagnosed. The prosthesis was exchanged for a homograft after 2 weeks, and he received a total of 8 weeks of benzyl-penicillin combined with 4 weeks of aminoglycoside, which produced a final eradication of the infection. Another patient, with a mechanical aortic valve prosthesis and aortic graft, had two relapses. He was first treated with benzyl-penicillin for 6 weeks combined with aminoglycoside for 2 weeks without surgery. Fifteen months post-treatment, a new IE episode was diagnosed. He received identical treatment to the first time with good clinical response, although 10 months later, a third episode was diagnosed. He underwent surgery on day 14 and received an aortic homograft followed by 6 weeks of benzyl-penicillin, and was finally cured. Two other patients were re-operated during antibiotic treatment, and one patient was re-operated 53 days after discharge because of prosthetic valve dehiscence. Except for the two patients with multiple episodes registered, none of the patients were registered again with IE of any type during the follow-up to February 2017.

## Discussion

This registry-based study presents the clinical characteristics, treatment, and outcome of 51 definite PVE cases caused by *P. acnes* and *Propionibacterium* spp. These anaerobic bacteria are unusual pathogens among patients with IE, and almost exclusively affect men with prosthetic valves. *Propionibacterium* spp. were the causative agent in approximately 8% of PVE cases reported to the SRIE. In a recent Swedish study focusing on patients with IE who underwent valvular surgery, 13% of IE in patients that had a prosthetic heart valve was caused by *Propionibacterium* spp. [13]. Both that study and our study display a much larger proportion of PVE cases being caused by *Propionibacterium* spp. than previously reported [8, 14]. The reason for this interesting finding could be the usage of a prolonged incubation protocol for selected blood cultures, a procedure that has been shown to more often diagnose *Propionibacterium* spp. [10, 14].

### Gender aspects

Notably in our study, 98% of patients were male. All patients with PVE caused by *P. acnes* were male; the only female patient had an infection with *P. avidum*. Male predominance has been reported in other studies of IE, but not at this level [15, 16]. Studies related to both shoulder surgery and cardiac surgery have shown that growth of *P. acnes* in tissue and skin samples is more common among males than females [17–19]. It is, thus, possible that cases of early IE are due to contamination of the surgical wound by the skin flora. However, the relation with late IE is less clear. Since men are reported to be colonized with *P. acnes* to a higher degree on the chest compared to women [18], men may be more prone to have transient bacteremia due to *P. acnes*.

### Risk factors

The majority of the cases in the present study had a long interval between valve surgery and infection. A plausible explanation could be that the PVE was caused by a hematologic route rather than by contamination during valve surgery. These findings are in concordance with previous studies [8, 10, 14]. There is still only limited knowledge about the frequency and relevance of bacteremia with *P. acnes*. Studies have shown that bacteremia can occur after endodontic treatment, both with *P. acnes* and with other bacteria originating from the oral mucous membranes [20, 21].

### Treatment and management

Most patients received treatment with beta-lactams; benzyl-penicillin in the majority of cases, partly in combination with aminoglycosides. *Propionibacterium acnes* is generally susceptible to benzyl-penicillin [22, 23] and, based on our results, it supports benzyl-penicillin as the drug of choice for the treatment of PVE caused by *Propionibacterium* spp. The patients who received vancomycin probably had this as part of the initial empiric treatment for PVE before the etiology was known.

### Prognosis/outcome

The outcome was favorable in the majority of cases. Sixty-three percent of the patients underwent cardiac surgery. Although two patients who were treated conservatively experienced relapses during follow-up, the majority of conservatively treated patients were cured, even one with a myocardial abscess displayed by transesophageal echocardiography. This shows that salvage is possible without surgery in selected cases, a question raised by Banzon et al. [14], whereas all their patients needed surgery. Follow-up after 6 months has been used to differentiate relapse from re-infection, suggesting that a new IE with onset later than 6 months after completion of treatment is a re-infection. A study using molecular diagnostic tools has shown this to be true in most cases, but since *Propionibacterium* spp. is slow-growing, there is a possibility that the patients in our study suffered relapse rather than re-infection [4].

Although in eight cases there was no registration of outcome at discharge, checking the SRIE showed that none of these patients had a new registered IE, which indicates they were cured. The mortality in the present study of IE caused by *P. acnes* or *Propionibacterium* spp. was 2.0%, which is lower than that reported for IE in general [24].

### Limitations

All departments of ID in Sweden are supposed to report cases of IE to the SRIE. The coverage of the SRIE is estimated to be 75–80%. The total number of cases reported annually to the registry is increasing; in recent years, 500–550 cases have been reported each year. However, the total number of IE episodes per year in Sweden is estimated to be about 650. There may be several reasons why cases are missed; patients are transferred between different departments, there is a lack of time for the registration procedure, or registration is simply forgotten.

There is always an uncertainty about patients who were not entered into the registry, and there may be a reporting bias. Since the registry is run by the doctors themselves, and used primarily for quality assurance or research, the risk of reporting bias ought to be low. One observation is that some departments of ID did not have any reported cases with IE caused by *Propionibacterium* spp. at all during this period, and, judging by the size of their referral area, they should have. The reason for this is unclear. It is possible that different microbiological laboratories have different protocols for cultures; for example, a shorter time before a culture is considered negative and, thus, a risk of overlooking a culture with a slow-growing *Propionibacterium* spp. This could mean that the IE is erroneously considered to be culture-negative.

At the Department of Laboratory Medicine, Clinical Microbiology, Örebro University Hospital, with a referral area of 275,000 inhabitants, we evaluated 114 positive blood culture bottles yielding *P. species* over approximately 3 years. Among five patients with six episodes of IE, there were 26 positive blood cultures with *P. acnes*. One patient with IE due to *P. avidum* had three positive blood cultures. The median time to positivity was 7 days (range: 4–15), and, so, the incubation of blood culture bottles for < 7 days may result in missed diagnosis of IE due to *P. acnes* (unpublished data).

## Conclusions

The present study shows that prosthetic valve endocarditis (PVE) caused by *Propionibacterium* spp. is a condition that almost exclusively affects men. Findings of *Propionibacterium* spp. in blood cultures in a patient with a prosthetic valve should alert the clinician to a possible diagnosis of infective endocarditis (IE). In patients with prosthetic heart valves, we recommend a protocol of prolonged incubation of blood cultures up to 14 days to minimize the risk of false-negative blood cultures. The prognosis was favorable, although a majority of patients required cardiac surgery during treatment. Benzyl-penicillin is the first-line antibiotic treatment option.
